# Bulbar onset amyotrophic lateral sclerosis with more evident symptoms in the left hemibody: a case report

**DOI:** 10.1093/omcr/omad045

**Published:** 2023-05-30

**Authors:** Manisha Chavan, Ajay Singh, Pinank K Bathvar, Sarang T Mehta, Abdul K Qadree, Sandesh Dhakal

**Affiliations:** Department of Medicine, Kakatiya Medical College, RangamPeta, Warangal, Telangana 506007, India; Department of Medicine, Sri Ram MurtiSmarak Institute of Medical Sciences, Bareilly, Uttar Pradesh 243202, India; Department of Medicine, SMT. NHL Municipal Medical College, Ahmedabad, Gujarat 380006, India; Department of Medicine, SMT. NHL Municipal Medical College, Ahmedabad, Gujarat 380006, India; Department of Medicine, Caribbean Medical University, Willemstad 4797, Curaçao; Department of Medicine, College of Medical Sciences, Bharatpur 44207, Nepal

## Abstract

Amyotrophic lateral sclerosis (ALS) is a progressive, degenerative neuromuscular condition. The procedure and difficulties involved in the clinical diagnosis of ALS have been the subject of numerous investigations. The understanding of the genetics and the epigenetics of the disease is still at infancy with several missing links. We present a case report of a 73-year-old woman suffering from bulbar onset ALS with a 4-month history of progressive dysphagia and dyspnea. She displayed tongue fasciculations and muscle atrophy. The bilateral palmomental reflexes, snout reflex, Hoffman, Babinski, diminished gag reflex, bilateral clonus and wild mood swings confirmed the neurodegenerative condition of the patient. The diagnosis of ALS can be challenging; therefore, the data presented may be useful to investigate its characteristics of the onset and to improve the understanding of the aspects of differentiation from other neurodegenerative disorders.

## INTRODUCTION

The neurological condition known as amyotrophic lateral sclerosis (ALS), commonly referred to as Lou Gehrig’s disease, or motor neuron disease (MND), is a progressive condition that affects the nerve cells that regulate voluntary muscles [1]. ALS is still considered one of the rare diseases and its incidence worldwide is 2.7–5 cases per 100 000 population annually. The patient’s growing weakening, manifested as muscular atrophy, hyperreflexia, fasciculations and muscle cramps, is the most striking feature of the disease. However, the sensory function remains unaffected [2]. We describe a case of ALS presenting bulbar onset with neurodegeneration, dysarthria and dysphagia.

## CASE REPORT

A 73-year-old woman with a history of arterial hypertension arrived at our neurology clinic complaining of speech difficulties and voice changes that had been going on for ⁓4 months. During the assessment, the patient displayed dysarthria and slurred speech. She had trouble swallowing both liquid and solid food and was constantly drooling. Her respiratory function was preserved. Upon examination, the patient displayed tongue fasciculations that were more noticeable on the left lateral side. The left hemibody showed more signs of atrophy in the thenar and hypothenar eminences, and the interosseous muscles of the hands. The biceps brachii and quadriceps femoris muscles both had clear fasciculations. The patient had a broad decline in muscle strength that scored 4/5 in the right hemibody and 3/5 in the left. The clinical examination revealed enhanced osteotendinous reflexes in the four limbs. The bilateral palmomental reflex was more pronounced on the left side. The patient also presented a snout reflex. In the left hemibody, Hoffman, Babinski and more obvious bilateral clonus were found. On pharyngeal examination, the gag reflex was not elicited, the left side palate was slightly dropped and the uvula showed deviation to the right. The differential and erythrocyte sedimentation rates on the hemogram were unaltered. Creatine phosphatase (CPK) measured 380 U/I. Nuclear Magnetic Resonance (NMR) scans of the skull and spine revealed no abnormalities of the brain or spinal cord. Fibrillations, positive sharp waves and fasciculations were detected spontaneously by electromyography in the investigated muscles (biceps, pronator teres, spinal cord and tibialis anterior). The notching and enhanced polyphasic amplitude of the motor unit potentials were visible.

In the differential diagnosis, entities that affected the first and second motor neurons were assessed from compressive causes in the stem and spinal cord, intoxications, demyelinating diseases and primary lateral sclerosis. Cerebrospinal fluid examination (CSF) showed an albumin-cytological dissociation with a protein of 151 mg/dl and the cell count of 2/mm^3^. CSF oligoclonal band study was negative. Acetyl cholinesterase receptor antibodies and anti-muscle specific kinase antibodies were negative, ruling out the possibility of myasthenia gravis. The serological testing of cytomegalovirus using enzyme linked immunosorbent assay for the immunoglobulin M (IgM) and immunoglobulin G (IgG) classes was negative. Thus diagnosis of bulbar onset ALS was made based on clinical, serological, CSF and electophysiological findings. The patient was put on symptomatic and supportive treatment with neuroprotective agents: Riluzol, Vitamin E and co-enzyme Q10. General measures were provided with gentle rehabilitation, amantadine and psychological support. At follow up visits, the patient had shown rapid functional deterioration and decline in life quality.

## DISCUSSION

ALS, referred to as classic MND, impacts motor neurons on both the upper and lower limbs. Typical bulbar onset ALS patients develop progressive limb symptoms within first 6 months. Patients with a duration of >20 months from bulbar onset to first significant limb involvement are more likely to have isolated bulbar palsy (IBP) [3]. Patients with bulbar onset ALS had poor prognosis and the disease course and survival time was significantly shorter. Early signs of bulbar onset ALS typically include bulbar muscles. Individuals gradually lose their strength, ability to speak, eat, move and even breathe, and practically all of the muscles they can control voluntarily become afflicted [4]. Within 2–3 years for cases of bulbar onset ALS and 3–5 years for patients with limb onset ALS, the paralysis progresses and results in death due to respiratory failure. [5]. Symptoms typically appear between the ages of 55 and 75, while the disease can strike at any age [6].

The patient in this case had both lower motor neuron (LMN) and upper motor neuron (UMN) bulbar signs. While Karam et al [7] found no evidence of a significant difference between UMN and LMN bulbar signs between the two groups, Burrel *et al*. [6] discovered that UMN bulbar signs were more prevalent in IBP.

ALS presents with significant clinical heterogeneity. The most common causes of diagnostic delays in ALS are frequent referrals to specialists, misdiagnosis, the location of disease onset, age at which it first manifests and comorbidities [4]. The trait of female gender predominance in bulbar onset ALS suggests that genetic factors may be involved. Most ALS cases have been described as sporadic, and more research on the pathophysiology and genetic susceptibility of ALS is required.

## Data Availability

All the available data has been presented in the manuscript.
