# Correction to: Bulbar onset amyotrophic lateral sclerosis with more evident symptoms in the left hemibody: a case report

**DOI:** 10.1093/omcr/omad077

**Published:** 2023-07-18

This is a correction to: Manisha Chavan and others, Bulbar onset amyotrophic lateral sclerosis with more evident symptoms in the left hemibody: a case report, Oxford Medical Case Reports, Volume 2023, Issue 5, May 2023, omad045, https://doi.org/10.1093/omcr/omad045

In the originally published version of this manuscript, there was a typographical error in the fifth author's name. This should read "Abdul K Qadree" instead of "Abdul K Qadri". This is emended in the article.

This error has been corrected (online).

